# The impact of sports event-brand fit on consumer brand responses: a meta-analytic review

**DOI:** 10.3389/fspor.2025.1598708

**Published:** 2025-06-23

**Authors:** Qingfeng Liu, Bing Liu

**Affiliations:** ^1^ School of Economics and Management, Shanghai University of Sport, Shanghai, China; ^2^ Physical Education College, Shanghai University, Shanghai, China

**Keywords:** sports event-brand fit, consumer cognitive responses, consumer emotional responses, consumer behavioral responses, meta-analysis, sports event sponsorship

## Abstract

**Introduction:**

In an era where global sports sponsorship is prevalent, companies aim to gain market share through sports sponsorship. Existing research typically examines this phenomenon by assessing consumer responses; however, different studies have yielded inconsistent or even contradictory findings.

**Methods:**

This study employs a meta-analytic approach to synthesize previous research, examining the impact of sports event-brand fit on consumers' cognitive, emotional, and behavioral responses, as well as the moderating effects of factors such as gender, sample source, and context type. The analysis includes 57 effect sizes from 31 studies, encompassing a total of 15,744 participants.

**Results:**

Sports event-brand fit has a positive effect on consumers' cognitive, emotional, and behavioral response. Moreover, sample sources and context types partially moderated these relationships, while gender showed no significant effect.

**Discussion:**

Findings suggest that sponsoring brands should align their sports sponsorships with specific developmental goals. Additionally, when sponsoring sports events in other countries, brands should respect cultural differences and tailor their marketing strategies to suit different contexts.

**Systematic Review Registration:**

https://www.prisma-statement.org

## 1 Introduction

Sponsorship is a vital channel in corporate marketing (1), and its effectiveness heavily depends on consumers' perceptions of sponsor–event fit (2, 3). Rooted in exchange theory, sponsorship fundamentally revolves around a reciprocal transaction process, wherein both sponsor and event organizers aim to achieve through sponsorship activities (4). It also constitutes a value co-creation process between sponsors and consumers: while companies invest resources to gain brand exposure, consumers construct brand meaning through their perception of fit (5).

Since the 1990s, research has increasingly focused on consumer-oriented sponsorship evaluation, particularly consumer brand responses (e.g., brand loyalty, purchase intention), to better understand the factors driving sponsorship success, as sponsorship benefits are often difficult to accurately quantify. However, research findings on the relationship between sport event-brand fit and consumer brand responses remain inconsistent (6). While high level sport event-brand fit is generally believed to generate positive consumer responses, some studies have challenged this assumption. For instance, Dos Santos et al. (3) failed to demonstrate that sponsors with a high level of fit with sports events received greater attention in eye-tracking experiments. Similarly, other studies (7–9) observed no direct positive effect of sport event-brand fit on brand awareness or trust. Conversely, Jagre et al. (10) found that appropriate sport event-brand incongruence could enhance consumer recall and attitudes.

These conflicting findings arises critical questions: Does a high level of fit between a sponsor and a sporting event necessarily yield the expected outcomes? Can corporate sponsorship of sports events always lead to significant positive brand responses? Furthermore, with increasing globalization, many companies sponsor international sports events—for example, the Swiss brand Rolex sponsors the Shanghai Masters, while the American brand Nike sponsors the Shanghai Marathon. However, consumer response is a complex and multidimensional psychological construct, influenced by cultural background and viewing context, which may shape brand responses to sponsorship fit in different ways (11, 12). These considerations underscore the need for a more systematic analysis of the relationship between sports event–brand fit and consumer brand responses.

Most of the existing studies in the literature tend to focus on the positive outcomes associated with sponsorship fit (13), leading to a relatively one-sided understanding of the relationship between sponsorship fit and consumer responses. In light of this, we aim to explore whether brand-event fit truly always leads to positive consumer brand responses. This meta-analytic approach is motivated by the need to systematically examine the impact of sponsorship fit across various consumer response dimensions, questioning whether the effect is universally positive or whether it varies under different conditions (e.g., cultural context, consumer characteristics, situation type).

To address these questions and bridge research gaps, this study employs a meta-analytic approach to quantitatively synthesize findings from existing studies, systematically categorizing different consumer brand responses and summarizing external factors such as cultural background and viewing context. Meta-analysis is a robust quantitative method that enables scholars to calculate and compare effect sizes from empirical studies, thereby providing a clearer understanding of the magnitude and variability of sponsorship effects (14). By identifying patterns and moderators, this study provides a comprehensive assessment of whether, and to what extent, sponsorship fit influences consumer brand responses. It also examines whether factors such as gender, culture, and in-person attendance play moderating roles in this relationship, offering more specific and valuable insights for companies in selecting sports sponsorships and designing marketing strategies.

## 2 Literature review and hypothesis development

### 2.1 Dimensions of consumer brand responses

According to information processing theory (3, 15), consumer brand responses represent the mapping of consumers' information cognition, emotions, and behaviors. They encompass a comprehensive evaluation of received information. In this study, corporate sports events sponsorship is seen as an information source, disseminated to consumers through communication channels, ultimately leading to evaluations of aspects such as brand recognition and emotions.

In sports sponsorship literature, consumer brand responses generally manifest as brand attitude, purchase intention, and more. These concepts are often collectively included in brand equity based on consumer assessments (16). For clearer classification, some scholars [such as (17)] reference the three-component attitude model (18) to divide consumer brand responses into consumers' cognition, affective, and behavioral intentions. This study is in the same way, categorizing consumer brand responses into three major categories and categorizing consumer psychological variables related to sponsors collected in sports event sponsorship research.

Furthermore, due to the inclusion of studies that involve various specific consumer brand response variables, collected variables have been categorized into three types of consumer brand responses based on defined attributes (see Table 1).

**Table 1:** Classification of consumer brand reactions.

Consumer brand responses	Research variables and their definitions
Consumer cognitive responses (CCR)	**Brand image (BI):** pertains to consumers' subjective cognitive judgments about the functional attributes and brand personality of the sponsored brand's products.
	**Perceived functional characteristics (PFC)**: due to the varied terminology used for similar concepts across different scholars' research, for the sake of consistency in this study, variables encompassing consumer perceptions of brand awareness, perceived quality, functional value, and other attributes are collectively referred to as perceived functional characteristics (PFC).
	**Perceived symbolic characteristics (PSC)** refer to consumers' perception of the extent to which a brand's association with a sporting event conveys symbolic meaning—such as brand identity, reputation, or alignment with social values—rather than merely offering functional benefits. Variables related to consumer perceptions of symbolic value, perceived corporate social responsibility, perceived goodwill, and other typical attributes of the sponsored brand are collectively referred to as perceived symbolic characteristics (PSC).
Consumer emotional responses (CER)	**Brand trust (BT)** reflects consumers' feelings of security, reliance, and trust in brands' inherent value. It demonstrates the emotional connection between consumers and brands.
	**Attitude toward brand (ATB**) represents an overall psychological response tendency formed towards the sponsors after exposure to sponsorship marketing stimuli. It encompasses evaluations like satisfaction, trust, agreement, and appreciation.
	**Brand association (BA)** refers to all elements connected to the brand in memory. These associations can take the form of abstractions, lifestyles, behaviors, or emotions. In sports sponsorship, an ideal brand association outcome is not just the linkage of the brand with the sports event but also the generation of positive emotional attitudes toward the sports event in the process.
Consumer behavioral intention (CBI)	**Brand loyalty (BL)** signifies a commitment that consumers have toward a brand. Compared to purchase intention, it implies a longer-term inclination toward certain behaviors.
	**Purchase intention (PI)** reflects consumers' inclination to purchase the sponsored brand. It serves as a determinant factor in consumer purchasing decisions.

### 2.2 The impact of sports event-brand fit on consumer brand responses

Despite the mixed findings in the literature, we hypothesize that sports event brand fit will generally have a positive impact on consumer brand responses. This hypothesis is based on the theoretical framework and the majority of empirical evidence supporting this relationship.

Like advertising, one of the primary objectives of sports sponsorship activities is to have the audience remember the brand name associated with the sponsored event. Therefore, there is significant interest in understanding the impact of sports sponsorship on the audience's ability to recognize or recall event sponsors. Furthermore, according to congruity theory (10, 19), perceived fit refers to the congruity between stimulus information on a specific topic and an individual's pre-existing knowledge structure (20). Consumers' perception of sponsor consistency with sponsored events aligns with their expectations of corporate marketing practices, increasing acceptance of the sponsorship (21). It has a more positive impact on the ability to identify and recall sponsoring brands. Conversely, inconsistency may increase the cognitive load on consumers, potentially making it challenging for participants to associate elements of the sports event with the sponsor. Several scholars have demonstrated the correlation between perceived fit and consumer brand cognitive responses [e.g. (22–24),]. Therefore, we propose the following hypothesis:

H1: Sports event-brand fit will positively affect consumer cognitive responses.

According to Schema Theory (25), individuals tend to evaluate external stimuli based on prior knowledge stored in relevant schemas, and they are more likely to possess stimuli that match their existing schemas than those with weaker associations (26, 27). The literature further specifies that the stored schema includes cognitive beliefs and affective elements (28). That is, the influence of the fit effect extends to one's affective domain, such as perceived value and attitude (29, 30). In this study, sports events represent well-developed schemas in consumers' knowledge networks. The fit between the images of the event and the sponsor, seen as symbolic associations between events and sponsors (31), can activate pattern connections and facilitate cognitive and affective transfer from sports events (e.g., enthusiasm and positive emotions) to the sponsoring brand. Specifically, the following hypothesis is proposed:

H2: Sports event-brand fit will positively affect consumer emotional responses.

Following Fishbein and Ajzen (18), consumer behavioral intentions are defined as the likelihood of an individual's behavior during the consumption process, indicating likelihood that consumers may take a particular action in the future. This behavioral intention is often susceptible to external stimuli or influences. The relationship between perceived fit and consumer behavioral intention is largely mediated through cognitive and emotional responses. The behavioral intentions of sponsorship tend to share common underlying mechanisms, such as attribution, congruence, and identification, which may also be associated with cognitive and affective outcomes (20, 32). For example, Koo et al. (17) confirmed the indirect impact of sponsor-event image congruence on associated purchase intentions through consumers' cognitive and affective responses. Perceived fit had a positive impact on attitudes toward sponsors, leading to increased purchase intentions among consumers (33), and might prove to be a valuable criterion for selecting sponsorship activities. In summary, numerous empirical studies have demonstrated considerable support for sponsorship effects on the conative outcome variable of intentions for favorable behaviors and purchases (15, 34). Based on these results, we propose the following hypotheses to explore the direct correlation between perceived fit and consumer behavioral intention:

H3: Sports event-brand fit will positively affect consumer behavioral intention responses.

### 2.3 The moderators between sports event-brand fit and consumer brand responses

Following the principles outlined by Kim et al. (35) for the selection of moderating variables, potential moderators included in this study were selected based on three criteria: firstly, the moderating effect of the factor must be logically reasonable or have an established theoretical foundation; Secondly, the factor can be coded based on previous research; Thirdly, there must be a sufficient number of studies available to ensure enough power to detect moderating effects. Therefore, we selected gender, culture, and situation type as moderators between sports event-brand fit and consumer brand responses. Mainly for the following three reasons.

Firstly, following Meyers-Levy (36), gender differences are due to differences in cognitive processing. In sports research, gender is a demographic segmentation variable. Previous studies have explored the relationship between consumer gender and sports marketing (37, 38). Secondly, understanding the role of culture in sponsorship is pivotal because a country's culture often shapes individuals' perceptions and behaviors (39), and substantial disparities exist between Eastern and Western cultures. Some cultures (such as Asian cultures) place more emphasis on collectivism and harmony, whereas others (such as North American and Northern European cultures) lean towards individualism (40). Lastly, the relationship between sports event-brand fit and consumer brand responses may also be moderated by situation type (spectator situation vs. non-spectator situation). According to information integration theory, consumer evaluations of brands or services can be influenced by situational factors (41). We categorized studies based on whether the participants were directly attending or watching the sports event (spectator) or if they were exposed to the sponsorship through other means such as media, advertisements, or other indirect methods (non-spectator). This classification helps in understanding the context in which the sponsorship impact was measured.

**Figure 1:**
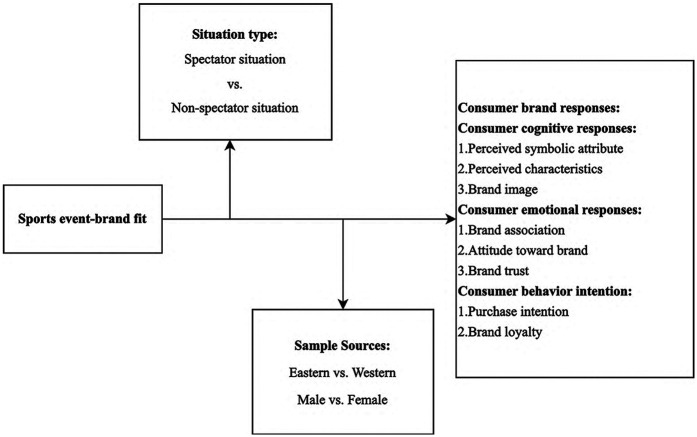
Research model.

Based on the above, we establish a research model framework (see Figure 1) and propose the following three hypotheses:

H4: The relationship between sports event-brand fit and consumer brand responses is moderated by gender. Specifically, for non-male consumers, sports event-brand fit has a stronger positive impact on consumer brand responses. Similarly, for male consumers, sports event-brand fit also has a weaker positive effect on consumer brand responses.
H5: The relationship between sports event-brand fit and consumer brand responses is moderated by sample sources. Specifically, for consumers in Western countries, sports event-brand fit has a stronger positive impact on consumer brand responses. Similarly, for consumers in Eastern countries, sports event-brand fit also has a weaker positive effect on consumer brand responses.
H6: The relationship between sports event-brand fit and consumer brand responses is moderated by situation type. Specifically, for consumers in spectator situations, sports event-brand fit has a stronger positive impact on consumer brand responses. Conversely, for consumers in non-spectator situations, sports event-brand fit has a weaker positive effect on consumer brand responses.

## 3 Method

We searched Chinese databases (CNKI and Wanfang) and English databases (Web of Science, EBSCO, and Google Scholar). We also used the following search terms in various combinations for the literature search: *sports sponsorship, sports sponsorship fit, sports event-brand fit, sports sponsorship congruence and sports sponsorship relevance.* The search was conducted for the period from 2000–2020. Additionally, to avoid missing relevant studies, a citation analysis was performed during the literature review process to identify and include additional relevant studies.

**Figure 2:**
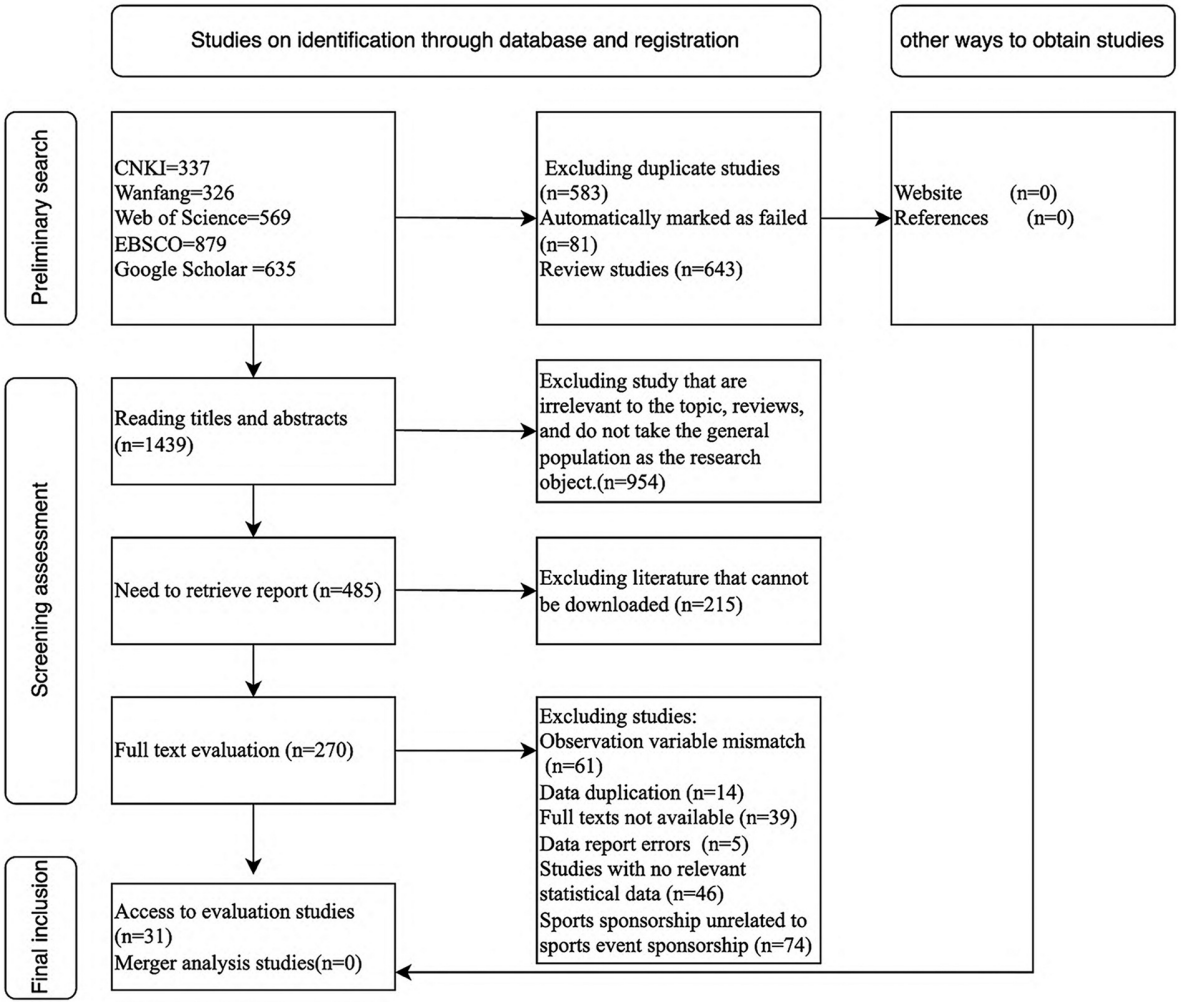
Flow diagram of study identification and selection.

EndNote X9 was used for literature selection. Studies were selected based on their focus on sports sponsorship specifically related to sports events, excluding other types of sports sponsorships such as sports venues and athletes (the encoding process is shown in Figure 2), as the impact of consistency in both cases on consumer attitudes differs (42). The final set of included studies consisted of 31 articles, encompassing a total of 57 effect sizes and involving 15,744 participants (see Supplementary Appendix A).

### 3.1 Coding procedure

We followed the suggestion of Lipsey and Wilson (43) and encoded each study with two main components: study characteristics and effect sizes. The first part, study characteristics, included information about the first author, publication year, publication type, sample nationality, sample size, proportion of male participants, etc. The second part, effect sizes, encompassed reliability, correlation coefficient, and significance level between sports event-brand fit and consumer brand responses.

Following expert advice and referring to Zhang (44), we compiled a meta-analysis literature quality evaluation scale, including the following criteria: Participant selection: random selection scores 2 points, non-random selection scores 1 point, and unreported scores 0 points. Data validity rate: data validity rate above 0.9 scores 2 points, between 0.8 and 0.9 scores 1 point, below 0.8 and unreported scores 0 points. Internal consistency reliability of measurement tools: reliability above 0.8 scores 2 points, between 0.7 and 0.8 scores 1 point, below 0.7 and unreported scores 0 points. Journal quality: SSCI (including SSCI-E) journals > CSSCI journals > Ordinary journals and unpublished papers are scores 2, 1, and 0 points, respectively. The final calculation results in a total score for each study, ranging from 0–8. A higher score indicates better literature quality.

The encoding process was conducted independently by two raters following the provided guidelines. In cases where inconsistencies arose, discrepancies were resolved by reviewing the original articles and engaging in discussions. The Kappa value for interrater reliability is 0.936, which exceeds 0.75 and is considered very good (45), indicating a high level of consistency between the two raters. For detailed information, please refer to AI.

### 3.2 Statistical methods

For studies where the correlation coefficient was not directly reported but other relevant statistics such as F, t, d, X^2^, mean (M), or standard deviation (SD) were provided, the corresponding correlation coefficient (r) was calculated using the formula as outlined by Card (46).

We employed the random-effects model, which assumes that the actual effects of studies might vary, and these differences are not only influenced by random error but also by variations in different samples (47). Furthermore, the appropriateness of selecting the random-effects model through a heterogeneity test (48).

We used Comprehensive Meta-Analysis Version 3 for conducting the primary effect test and moderation effect analysis. Moderators were analyzed in two forms: When the moderator is continuous, a meta-regression analysis is used to examine whether the results are significant about the moderator; When the moderator is categorical, subgroup analysis is employed to test the significance of the results within different subgroups.

## 4 Results

### 4.1 Publication bias test

The funnel plot displays a concentration of effect sizes above the funnel and a uniform distribution on both sides of the overall effect. The results of Egger's linear regression are not significant, with an intercept of 2.056 and a 95% confidence interval of [−2.907, 7.02], which includes 0. The *p*-value is 0.41. These findings suggest that this study has no significant publication bias, and the meta-analysis estimation results are relatively reliable.

### 4.2 Heterogeneity test

The test results reveal a *Q*-value was 2954.344 (*p* < .001), and the *I*^2^ value was 98.172%, exceeding the 75% rule proposed by Higgins et al. (48). It indicates significant heterogeneity among the results. Therefore, employing a random-effects model for further analysis is appropriate. Additionally, these findings suggest that differences in estimates between different studies may be interfered with by various research characteristic factors. Exploring potential moderators that could impact the relationship between sports event-brand fit and consumer brand responses is warranted.

### 4.3 Results between sports event-brand fit and consumer cognitive responses

The results (see Table 1) demonstrate that sports sponsorship fit has a significant overall effect on consumer cognitive responses (*ρ* = .506, *p* < .001). Specifically, it shows a positive correlation with perceived functional characteristics (*ρ* = .68, *p* < .001), a positive correlation with brand image (*ρ* = .342, *p* = .023 < .05), and no significant impact on perceived symbolic characteristics (*p* = .119). Therefore, H1 is supported.

Regarding the results of sports event-brand fit on consumer emotional responses, it is evident that sports event-brand fit can induce changes in consumer emotional responses towards the sponsors, positively influencing their emotions (*ρ* = .422, *p* < .001). In particular, sports event-brand fit significantly enhances attitude toward the brand (*ρ* = .491, *p* < .001), brand trust (*ρ* = .423, *p* < .001), and brand associations (*ρ* = .767, *p* = .011 < .05). Hence, H2 is supported.

Sports event-brand fit can encourage consumers' purchase behavior towards the sponsored brand (*ρ* = .602, *p* < .001). Specifically, it significantly promotes purchase intention (*ρ* = .541, *p* < .01) and brand loyalty (*ρ* = .744, *p* < .01). Therefore, H3 is supported.

### 4.4 Moderators analyses

The within-group heterogeneity test statistic Q_W_ for the relationships between sports event-brand fit and consumer cognitive responses, affective responses, and behavioral intention responses (see Table 2) are all significant, indicating the presence of potential moderators in these relationships. Subsequently, this study conducted moderation effect tests.

**Table 2:** Meta-analysis of the impact of sports event-brand fit on consumer brand responses.

Dependent variable	k	N	*ρ*	95% CI		Two-Tailed Tests		Q_W_	*I* ^2^
				LL	UL	Z	P		
CCR	20	8,079	0.506	0.332	0.647	5.137	0.000	1,648.470***	98.847
PSC	6	2,945	0.380	−0.103	0.718	1.559	0.119	597.578***	99.163
PFC	8	2,504	0.680	0.386	0.848	3.853	0.000	638.543***	98.904
BI	6	2,630	0.342	0.048	0.582	2.267	0.023	300.531***	98.336
CER	23	7,866	0.514	0.422	0.595	9.477	0.000	575.074***	96.174
ATB	13	4,149	0.491	0.400	0.574	9.175	0.000	150.933***	92.049
BT	5	1,900	0.423	0.272	0.554	5.114	0.000	57.506***	93.044
BA	3	488	0.767	0.228	0.946	2.540	0.011	115.314***	98.266
CBI	12	3,702	0.602	0.413	0.741	5.318	0.000	655.413***	98.322
PI	8	2,664	0.541	0.264	0.735	3.543	0.001	493.878***	98.580
BL	3	811	0.744	0.370	0.911	3.290	0.001	132.495***	98.437

**Note(s):** In the provided information, the symbols have the following meanings: k represents the number of effect sizes; N represents the total sample size; ρ denotes the corrected overall correlation coefficient; the 95% confidence interval provides the range for the corrected overall correlation coefficient; two-tailed tests report *Z*-values and *p*-values; Q_W_ is the within-group heterogeneity test statistic; I^2^ reflects the proportion of total variance in effect sizes that can be attributed to heterogeneity.

CCR, consumer cognitive responses; PSC, perceived symbolic characteristics; PFC, perceived functional characteristics; BI, brand image; CER, consumer emotional responses; ATB, attitude toward brand; BT, brand trust; BA, brand association; CBI, consumer behavioral intention; PI, purchase intention; BL, brand loyalty.

*** is *p* < .001.

Firstly, a meta-analysis was performed for the continuous moderator (proportion of male participants). The results indicate that gender does not significantly moderate the relationships between sports event-brand fit and consumer cognitive response [95% CI (−1.293, 2.144), *p* = .628], affective response [95% CI (−0.838, 1.949), *p* = .435], and behavioral intention response [95% CI (−1.391, 0.912), *p* = .684]. Hence, H4 is not supported. The moderating effects of categorical variables (situation type, sample source), as depicted in Table 3.

**Table 3:** The moderating effect of moderators on the relationship between sports event-brand fit and consumer brand responses.

Dependent variable	Moderators		k	N	ρ	95% CI		Q_W_	Q_B_	*I* ^2^
						LL	UL			
CCR	Sample Source	Eastern	13	4,975	0.493***	0.472	0.514	1,026.878***	2.539	98.831
		Western	7	3,104	0.465***	0.437	0.492	619.053***		99.031
	Situation type	Spectator situation	8	2,735	0.421***	0.390	0.452	149.940***	24.112***	95.311
		Non-spectator situation	12	5,344	0.512***	0.492	0.532	1,474.418***		99.254
CER	Sample Source	Eastern	14	4,792	0.503***	0.481	0.524	433.776***	4.615*	97.003
		Western	9	3,074	0.464***	0.436	0.492	136.683***		94.147
	Situation type	Spectator situation	8	1,598	0.496***	0.458	0.533	31.049***	0.200	77.455
		Non-spectator situation	11	5,357	0.486***	0.466	0.507	542.785***		98.158
CBI	Sample Source	Eastern	8	2,937	0.547***	0.521	0.572	473.843***	77.868***	98.523
		Western	4	765	0.751**	0.718	0.781	103.702***		97.107
	Situation type	Spectator situation	6	1,761	0.553***	0.520	0.585	165.803***	17.534***	96.984
		Non-spectator situation	5	1,714	0.645***	0.616	0.672	470.542***		99.150

**Note(s):** In the provided information, the symbols have the following meanings: k represents the number of effect sizes; N represents the total sample size; ρ denotes the corrected overall correlation coefficient; the 95% confidence interval provides the range for the corrected overall correlation coefficient; two-tailed tests report *Z*-values and *p*-values; Q_W_ is the within-group heterogeneity test statistic; Q_B_ is the between-group heterogeneity test statistic; I^2^ reflects the proportion of total variance in effect sizes that can be attributed to heterogeneity.

* is *p* < .05.

** is *p* < .01.

*** is *p* < .001.

Secondly, consumer cognitive responses show no significant difference across sample nations (Q_B_ = 2.539, *p* = .111). However, consumer emotional responses show a significant difference across sample nations (Q_B_ = 4.615, *p* < .05), with the effect size in Eastern culture (*ρ* = .503) being greater than in Western culture (*ρ* = .464). Similarly, there is a significant difference in consumer behavioral intention across sample nations (Q_B_ = 77.868, *p* < .001), with the effect size in Eastern culture (*ρ* = .547) being weaker than in Western culture (*ρ* = .751). Therefore, H5 is partially supported.

Finally, the moderating effect of situation type on the relationship between sports event-brand fit and consumer brand responses was examined. Consumer emotional responses (Q_B_ = 0.200, *p* = .655) show no significant difference between spectator and non-spectator situations. However, consumer cognitive responses (Q_B_ = 50.243, *p* < .001) and consumer behavioral intention (Q_B_ = 17.534, *p* < .001) show significant differences between spectator and non-spectator situations. Interestingly, effect sizes for participants in the spectator situations (*ρ*_CCR_ = .421, *ρ*_CBI_ = .547) are weaker than those in the non-spectator situations (*ρ*_CCR_ = .512, *ρ*_CBI_ = .751). Overall, H6 is partially supported.

## 5 Discussion

### 5.1 The relationship between sports event-brand fit and consumer brand responses

Although there have been meta-analytic studies on the relationship between sports event-brand fit and consumer brand responses, none have provided a detailed classification of the various consumer response dimensions. We employed meta-analysis to synthesize existing findings and estimate the relationship between sports event-brand fit and different types of consumer brand responses. Our results reveal that the impact of sports event-brand fit varies across distinct dimensions of consumer response, such as attitudes, behaviors, and emotional connections. This suggests that the effectiveness of sponsorships is not uniform but depends on the specific consumer response being targeted. These findings offer practical insights for marketers, highlighting the importance of aligning sponsorship strategies with the particular consumer response dimension they aim to influence.

Specifically, our analysis reveals that sports event-brand fit has a positive influence on consumer brand perception in terms of cognitive responses. However, we did not observe a significant effect on perceived symbolic characteristics. Our findings, which indicate a lack of significant relationship between sport event brand fit and symbolic features, contribute to the ongoing debate in the literature. This result aligns with studies like Simmon et al. (22) but contrasts with others that support a positive relationship. These mixed results highlight the complexity of this relationship and suggest that further research is needed to clarify the conditions under which sport event brand fit influences symbolic features.

On the one hand, consumers' psychology and behavior are indeed complex and nuanced, particularly concerning perceived symbolic features. Establishing a profound and enduring presence in consumers' minds requires sustained marketing efforts over time. While sports event sponsorship can facilitate the transfer of the event's image to the brand (49), this process necessitates patience and repeated sponsorships. Research suggests that long-term sponsorships are more conducive to consumers' recall and recognition of sponsoring brands (22). Therefore, it is understandable that high-fit sports sponsorship may not always lead to immediate improvements in perceived symbolic features. Instead, achieving positive outcomes in this domain may require ongoing investment and consistent brand exposure over time. This finding underscores the importance of adopting a strategic and long-term approach to sports event sponsorship for companies seeking to enhance their brand perceptions among consumers.

In examining the relationship between sports event-brand fit and consumer emotional responses, we find that the correlation between sports event-brand fit and brand associations is particularly strong (*ρ* = .767), a finding consistent with previous research by Rifon et al. (50). This close linkage between sponsored events and sponsors can create robust associative memories in the minds of the audience, enhancing consumers' trust and recognition of sponsors.

We find that the correlation between sports event-brand fit and brand trust is 0.423. Brand trust places higher emotional demands on consumers, as it emphasizes their affective reliance on brands. As highlighted by Morgan et al. (51), trust between consumers and brands fundamentally involves a social exchange relationship. Brands pledge to deliver emotional connection, reliance, and enriched meaning to consumers, who reciprocate by investing emotional resources through trust and establishing a sustained relationship. Thus, achieving consumer brand trust in sports event sponsorship necessitates brands demonstrating, through sponsorship activities, their consistent provision of meaningful and positive emotional resources to consumers, both presently and in the future. This deeper and enduring emotional interaction between brands and consumers is crucial for fostering brand trust, which imposes higher demands on brands in their interactions with consumers. While high-fit sports event sponsorship can indeed induce consumer brand trust, achieving higher levels of consumer emotional responses may require complementary marketing measures to further enhance brand-added value.

### 5.2 The effect of moderators

Firstly, the moderating effect of gender on the relationship between sports event-brand fit and consumer brand responses is found to be non-significant. This suggests that there might be cross-gender stability in this relationship, indicating consistent perceptions and evaluations across genders. Empirical research findings have indeed shown that gender differences are not significant in terms of how brand associations influence consumer brand evaluations. This implies that men's and women's evaluations of sponsors remain consistent within the sponsorship relationship.

Secondly, the moderating effect of sample sources on the relationship between sports event-brand fit and consumer brand responses reveals interesting cultural nuances. Firstly, there are no significant differences in the impact of sports event-brand fit on consumer cognitive responses across sample nations. This finding aligns with the trend of international brand development, where sports sponsorship transcends international boundaries. Therefore, consumers from different countries exhibit consistent cognitive responses to sports event sponsorship information. Moving on to consumer emotional responses, the correlation is found to be higher for Eastern cultures compared to Western ones. This discrepancy reflects the cultural differences between Eastern collectivism and Western individualism. Eastern cultures, emphasizing harmony and consistency, tend to positively respond to sponsor alignment with sports events, meeting consumers' psychological expectations and eliciting more favorable emotional responses towards the brand. In contrast, for consumer behavioral responses, the effect value is weaker for Eastern cultures compared to Western ones. This observation underscores the cultural disparities in consumer behavior and market maturity between Eastern and Western countries, while also validating that cultural sensitivity is a fundamental principle in international marketing. It not only helps to strengthen resonance with local consumers but also mitigates risks stemming from brand inconsistency or negative perceptions. Despite the rapid development of the Eastern sponsorship market, Western consumers demonstrate higher behavioral intentions in response to high levels of sports event-brand fit, possibly due to the more established sports sponsorship landscape in Western countries.

Lastly, the moderating effect of situation type on the relationship between sports event-brand fit and consumer brand responses can be summarized as follows.

Situation type moderates the relationship between sports event-brand fit and consumer cognitive and behavioral responses, with a stronger effect observed in non-spectator situations compared to spectator situations. This underscores the significance of off-site sponsorship advertising strategies. Due to the limited space for sponsorship information within sports events' central operations, sponsors often resort to placing logos on the field. However, this association between sponsors and sports events may not be readily apparent to consumers inside the venue, leading to uncertainty. In contrast, outside the sports event venue, sponsorship advertising enables companies to convey the essence of the sponsorship relationship more clearly to the target audience. This enhanced understanding reinforces the correlation between sports event-brand fit and consumer cognitive and behavioral responses.

Situation type does not moderate the relationship between sports event-brand fit and consumer emotional responses. Although viewers experience emotional fluctuations while watching a game, their emotional attachment to the sports event and their primary purpose for watching are long-term and stable, driven by their love for the sports event, and these emotions are not influenced by specific context (52). Therefore, their emotions towards sponsors remain equally stable across different contexts.

## 6 Practical implications

In light of the intricate relationship between sports event-brand fit and consumer brand responses, this study offers valuable insights for businesses involved in event sponsorship marketing. Aligning with brand development goals and stage is crucial. Considering that sponsoring brands may be at different stages of development, they should align their sports sponsorship with specific development goals. In the initial stages, emphasis can be placed on establishing brand image and fostering emotional connections with consumers. For example, if Brand X is a health and fitness brand, sponsoring a marathon or fitness expo may enhance consumer brand awareness and emotional engagement. Brand X can also leverage social media campaigns to highlight the narratives of sponsored athletes or events, thereby augmenting the brand's symbolic value. Regarding sponsorship duration and involvement in multiple sponsorships, it's essential to contemplate both factors. Through long-term and multiple sponsorships, brands can develop a more comprehensive brand response network among consumers, thereby enhancing brand awareness and affinity. Respecting cultural differences is imperative, especially concerning brand internationalization. Brands may choose cross-border sponsorship of sports events to increase international influence. However, it is crucial for brands to show due respect for cultural differences, ensuring that sponsorship activities yield positive effects across diverse countries. Tailor marketing strategies for different contexts is vital. In live event scenarios, priority should be given to integration with sports event elements and emotional engagement with on-site audiences. For non-spectators, managers should focus on media partnerships, digital advertising, and influencer collaborations that extend the sponsorship's reach beyond the event itself. These recommendations aim to assist brands in effectively navigating sports event sponsorships, resulting in enhanced brand responses and market impact. Managers should continually assess the effectiveness of sports event sponsorships through consumer feedback and engagement metrics. Surveys, social media sentiment analysis, and sales data can provide insights into consumer responses to sponsorships. Sponsoring brands can use this data to refine their sponsorship strategies, ensuring they align with consumer preferences and maximize return on investment.

## 7 Limitations and future research

This study, while providing valuable insights into the relationship between sports event-brand fit and consumer brand responses, has several limitations that warrant discussion. Firstly, although the meta-analytic methodology necessitates comprehensive inclusion of existing study data, certain unpublished literature may have been challenging to retrieve. Despite our extensive search across multiple academic databases, potential data omissions could exist, which may impact the generalizability of the findings. Future research could benefit from efforts to include grey literature, such as conference papers or dissertations, to further minimize publication bias. Secondly, this study categorized consumer brand responses into distinct dimensions (e.g., attitudes, behaviors, and emotional connections). However, as research progresses, scholars have increasingly refined the concept of sports sponsorship fit into various sub-dimensions, such as image fit and target fit (53). Future studies could explore the differential impacts of these specific fit dimensions on consumer brand responses, offering more granular insights into how distinct aspects of fit influence consumer perceptions and behaviors. Thirdly, our research primarily focused on the effects of sports event-brand fit, while other forms of sports sponsorship, such as athlete endorsements, venue naming rights, or team sponsorships, were not examined. Subsequent research could expand this scope by comparing the effects of different sponsorship types on consumer brand responses, thereby providing a more nuanced understanding of the strategic considerations for various sponsorship arrangements. Fourthly, while we examined sample sources, gender, and Situation type as moderating variables, we used a binary approach by categorizing sample sources as Eastern and Western cultures. Given the diversity of cultures, future research could adopt a multidimensional approach to categorize sample sources, enhancing the cultural sensitivity and theoretical depth of the research. In addition, there are many potential moderating variables worth exploring, such as consumers' income levels, event types, sponsor brand types, and sponsorship duration, to provide a more comprehensive understanding of sponsorship effectiveness. Finally, this study did not consider broader contextual factors, such as perceptions of sponsor motives, moral judgments of sponsorship associations (e.g., gambling or alcohol sponsors), or the influence of opposition team fans. Future research could incorporate these contextual variables to enrich the understanding of how sponsorship effectiveness varies under different circumstances. Additionally, future research could also examine longitudinal sponsorship effects to assess how symbolic or loyalty responses evolve over time. With the rise of digital and virtual platforms (e.g., esports or online events) (54), it is also worthwhile to explore how brand-event fit functions in these new sponsorship contexts. Moreover, investigating brand risk mitigation strategies in cases of poor fit can offer practical guidance for sponsors navigating challenging or incongruent associations. By addressing these limitations, future studies can further advance the understanding of sports sponsorship effectiveness, providing both theoretical and practical insights for academics and practitioners alike.

## 8 Conclusion

Through meta-analysis, we find that: overall, sports event-brand fit positively impacts consumer brand cognitive responses, emotional responses, and behavioral intention responses. However, specific consumer responses, such as perceived symbolic characteristics and brand associations, may not be significantly affected; The impact of sports event-brand fit on different consumer brand responses varies, suggesting that positive consumer brand responses are not instantaneous. For instance, achieving consumer brand responses like perceived symbolic characteristics and brand loyalty necessitates deeper and more prolonged interaction between brands and consumers through sports event-brand fit; The relationship between sports sponsorship fit and consumer brand emotional and behavioral responses is moderated by sample sources. High-level sports event-brand fit is more advantageous for generating positive brand emotional responses among consumers in Eastern countries. Conversely, it is more effective in enhancing behavioral intention responses among consumers in Western countries; Situation type also moderates the relationship between sports event-brand fit and consumer brand responses. High-level sports event-brand fit is more beneficial for generating positive cognitive and behavioral responses among off-site spectators. This study deepens our understanding of the relationship between sports event sponsorship fit and consumer responses, emphasizing that brand-event consistency not only has a positive impact but also highlights more complex and diverse outcomes, filling a gap in previous research that has been less explored. Furthermore, this study reveals the influence of sports event brand consistency on consumer responses in different cultural contexts and viewing situations, providing practical insights for marketers in various international markets.

## Data Availability

The original contributions presented in the study are included in the article/Supplementary Material, further inquiries can be directed to the corresponding author.
