# Supplementary material for: The impact of sports event-brand fit on consumer brand responses: a meta-analytic review
Source: Front Sports Act Living. 2025 Jun 23;7:1598708. doi: 10.3389/fspor.2025.1598708 (PMC12230083; doi:10.3389/fspor.2025.1598708)
Supplement: Supplementary file 1 [file Table1.docx]

Supplementary Material

# Supplementary Tables

**Appendix A** The basic information of the original studies included in the meta-analysis.

| Studies | Sample size | Gender | Situation type | Sample source | Fit measure | Brand responses | Correlation coefficient | Literature quality assessment |
| --- | --- | --- | --- | --- | --- | --- | --- | --- |
| Aguiló-Lemoine, À. E., 2020 | 42 | 0.876 | spectator | Spain | Congruence | CCR | 0.572 | 8 |
| Alguacil, M., 2020 | 307 | 0.404 | non-spectator | Spain | Congruence | CER | 0.55 | 6 |
| Baek, W. Y., 2017 | 382 | 0.743 | spectator | Korea | Fit | CBI | 0.228 | 7 |
| Bao C C, 2011 | 507 | 0.402 | non-spectator | China | Congruence | CER | 0.19 | 5 |
| Coelho, M. G. R., 2019 | 1973 | 0.514 | non-spectator | Brazil | Fit | CCR | 0.525 | 6 |
| Demirel, A., 2020 | 280 | 0.693 | non-spectator | USA | Fit | CCR | 0.69 | 8 |
| Demirel, A., 2020 | 280 | 0.693 | non-spectator | USA | Fit | CBI | 0.76 | 8 |
| Dos Santos, M. A., 2019 | 240 | 0.463 | spectator | Chile | Congruence | CCR | 0.187 | 6 |
| Dos Santos, M. A., 2019 | 21 | 0.463 | spectator | Chlie | Congruence | CER | 0.319 | 8 |
| Dos Santos, M. A., 2019 | 21 | 0.463 | spectator | Chile | Congruence | CBI | 0.178 | 8 |
| Fan W P, 2008 | 280 | 0.41 | non-spectator | China | Match | CER | 0.535 | 4 |
| Fan W P, 2008 | 280 | 0.41 | non-spectator | China | Match | CBI | 0.603 | 4 |
| Fan W P, 2008 | 280 | 0.41 | non-spectator | China | Match | CCR | 0.629 | 4 |
| Groza, M. D., 2012 | 106 | 0.51 | non-spectator | USA | Congruence | CCR | 0.473 | 8 |
| Khuong, M., 2017 | 227 | 0.714 | - | Vietnam | Fit | CER | 0.532 | 3 |
| Khuong, M., 2017 | 227 | 0.714 | - | Vietnam | Fit | CBI | 0.543 | 3 |
| Liu, H., 2015 | 309 | 0.427 | spectator | China | Congruence | CER | 0.484 | 6 |
| Liu, H., 2015 | 309 | 0.427 | spectator | China | Congruence | CCR | 0.352 | 6 |
| Liu, H., 2015 | 251 | 0.347 | spectator | Korea | Congruence | CER | 0.48 | 6 |
| Liu, H., 2015 | 251 | 0.347 | spectator | Korea | Congruence | CCR | 0.457 | 6 |
| Li J J, 2016 | 879 | 0.561 | non-spectator | China | Match | CCR | 0.189 | 6 |
| Liu Q F, 2020 | 127 | 0.354 | spectator | China | Fit | CER | 0.632 | 4 |
| Liu Q F, 2020 | 127 | 0.354 | spectator | China | Fit | CCR | 0.088 | 4 |
| Mazodier, M., 2014 | 449 | 0.494 | non-spectator | France | Fit | CER | 0.22 | 6 |
| Mo L, 2009 | 180 | 0.428 | non-spectator | China | Match | CER | 0.249 | 4 |
| Mo L, 2009 | 180 | 0.428 | non-spectator | China | Match | CCR | 0.278 | 4 |
| Mo L, 2009 | 280 | 0.428 | non-spectator | China | Match | CCR | 0.261 | 4 |
| Olson, E. L., 2010 | 1149 | 0 | non-spectator | Norway and Greece | Fit | CER | 0.37 | 7 |
| Papadimitriou, D., 2016 | 127 | 0.47 | spectator | Greece | Fit | CER | 0.24 | 6 |
| Papadimitriou, D., 2016 | 127 | 0.47 | spectator | Greece | Fit | CCR | 0.32 | 6 |
| Papadimitriou, D., 2016 | 127 | 0.47 | spectator | Greece | Fit | CBI | 0.3 | 6 |
| Park, J. Y., 2020 | 202 | 0.535 | spectator | Indonesia | Congruence | CER | 0.454 | 4 |
| Park, J. Y., 2020 | 202 | 0.535 | spectator | Indonesia | Congruence | CCR | 0.355 | 4 |
| Park, J. Y., 2020 | 202 | 0.535 | spectator | Indonesia | Congruence | CBI | 0.501 | 4 |
| Park, S., 2018 | 151 | 0.77 | spectator | Korea | Fit | CER | 0.314 | 6 |
| Park, S., 2018 | 151 | 0.77 | spectator | Korea | Fit | CCR | 0.275 | 6 |
| Peng J, 2020 | 70 | 0.443 | non-spectator | China | Match | CER | 0.312 | 5 |
| Peng J, 2020 | 70 | 0.443 | non-spectator | China | Match | CER | 0.264 | 5 |
| Rui Biscaia, 2018 | 228 | - | - | Brazil | Congruence | CER | 0.47 | 6 |
| Rui Biscaia, 2018 | 161 | - | - | Brazil | Congruence | CER | 0.47 | 6 |
| Rui Biscaia, 2018 | 293 | - | - | Brazil | Congruence | CER | 0.46 | 6 |
| Seok Sohn, Y., 2012 | 304 | 0.5 | non-spectator | Korea | Fit | CCR | -0.292 | 8 |
| Shi J H, 2011 | 630 | 0 | non-spectator | China | Congruence | CER | 0.295 | 7 |
| Shi J H, 2011 | 630 | 0 | non-spectator | China | Congruence | CBI | 0.128 | 7 |
| Sung, M., 2016 | 685 | 0 | spectator | Korea | Fit | CCR | 0.58 | 8 |
| Sung, M., 2016 | 685 | 0 | spectator | Korea | Fit | CBI | 0.64 | 8 |
| Tu C W, 2016 | 344 | 0.901 | spectator | China | Congruence | CBI | 0.338 | 4 |
| Uhrich, S., 2014 | 336 | 0.485 | non-spectator | Germany | Congruity | CCR | -0.491 | 7 |
| Wan C L, 2010 | 1261 | 0 | non-spectator | China | Congruence | CER | 0.449 | 6 |
| Wan C L, 2010 | 1261 | 0 | non-spectator | China | Congruence | CCR | 0.347 | 6 |
| Wang, M. C. H., 2017 | 410 | 0.624 | spectator | Taiwan | Congruence | CER | 0.45 | 7 |
| Zaharia, N., 2016 | 337 | 0.85 | non-spectator | USA | Fit | CER | 0.69 | 6 |
| Zaharia, N., 2016 | 337 | 0.85 | non-spectator | USA | Fit | CBI | 0.65 | 6 |
| Zhang C, 2013 | 187 | 0.561 | non-spectator | China | Match | CCR | 0.788 | 5 |
| Zhang C, 2013 | 187 | 0.561 | non-spectator | China | Match | CCR | 0.77 | 5 |
| Zhang C, 2013 | 187 | 0.561 | non-spectator | China | Match | CER | 0.777 | 5 |
| Zhang C, 2013 | 187 | 0.561 | non-spectator | China | Match | CBI | 0.759 | 5 |

**Note(s):** Y is spectator situation; N is non- spectator situation; - is date missing.
